# Supplementary material for: Maternal Diet Quality and the Health Status of Newborns
Source: Foods. 2022 Dec 2;11(23):3893. doi: 10.3390/foods11233893 (PMC9739031; doi:10.3390/foods11233893)
Supplement: Supplementary file 1 [file foods-11-03893-s001.zip › foods-1949104-supplementary.pdf]

Supplementary materials

Table S1: 67 substances belonging to five groups of persistent organic pollutants (POPs) measured in food samples.

Polychlorinated biphenyls **PCBs**			
2,4,4'-Trichlorobiphenyl	**PCB 28**	2,2',3,4,4',5'-Hexachlorobiphenyl	**PCB 138**
2,2',5,5'-Tetrachlorobiphenyl	**PCB 52**	2,2',4,4',5,5'-Hexachlorobiphenyl	**PCB 153**
2,2',4,5,5'-Pentachlorobiphenyl	**PCB 101**	2,2',3,3',4,4',5-Heptachlorobiphenyl	**PCB 170**
2,3',4,4',5-Pentachlorobiphenyl	**PCB 118**	2,2',3,4,4',5,5'-Heptachlorobiphenyl	**PCB 180**
**Organochlorine pesticides OCPs**			
p,p'- Dichlorodiphenyldichloroethylene	**p,p'-DDE**	Hexachlorobenzene	**HCB**
o,p'- Dichlorodiphenyldichloroethylene	**o,p'-DDE**	α-Hexachlorocyclohexane	**α-HCH**
p,p'- Dichlorodiphenyltrichloroethane	**p,p'-DDT**	β-Hexachlorocyclohexane	**β-HCH**
o,p'- Dichlorodiphenyltrichloroethane	**o,p'-DDT**	γ-Hexachlorocyclohexane	**γ-HCH**
p,p'- Dichlorodiphenyldichloroethane	**p,p'-DDD**		
o,p'- Dichlorodiphenyldichloroethane	**o,p'-DDD**		
**Brominated flame retardants BFRs**			
2,4,4′-tribromodiphenyl ether	**PBDE 28**	α-Hexabromocyclododecane	**α-HBCD**
2,2′,4,4′-tetrabromodiphenyl ether	**PBDE 47**	β-Hexabromocyclododecane	**β-HBCD**
2,2′,4,4′,5-pentabromodiphenyl ether	**PBDE 99**	γ-Hexabromocyclododecane	**γ-HBCD**
2,2′,4,4′,6-pentabromdifenylether	**PBDE 100**	Pentabromophenol	**PBP**
2,2',4,4',5,5′-hexabromodiphenyl ether	**PBDE 153**		
2,2′,3,4,4',5',6-heptabromodiphenyl ether	**PBDE 183**		
Decabromodiphenyl ether	**PBDE 209**		
**Perfluorinated alkylated substances PFAS**			
Perfluoro-*n*-butanoic acid	**PFBA**	Perfluoro-1-butanesulfonate	**PFBS**
Perfluoro-*n*-pentanoic acid	**PFPeA**	Perfluoro-1-hexanesulfonate	**PFHxS**
Perfluoro-*n*-hexanoic acid	**PFHxA**	Perfluoro-1-octanesulfonate	**PFOS**
Perfluoro-*n*-heptanoic acid	**PFHpA**	Perfluoro-1-decanesulfonate	**PFDS**
Perfluoro-*n*-octanoic acid	**PFOA**		
Perfluoro-*n*-nonanoic acid	**PFNA**		
Perfluoro-*n*-decanoic acid	**PFDA**		
Perfluoro-*n*-undecanoic acid	**PFUdA**		
Perfluoro-*n*-dodecanoic acid	**PFDoA**		
Perfluoro-n-tridecanoic acid	**PFTrDA**		
Perfluoro-n-tetradecanoic acid	**PFTeDA**		
**Polycyclic aromatic hydrocarbons PAHs**			
5-Methylchrysene	**5MC**	Cyklopenta[c,d]pyrene	**CPP**

Acenaphthene	**ACE**	Dibenz[a,h]anthracene	**DBahA**
Acenaphthylene	**ACY**	Dibenzo[a,e]pyrene	**DBaeP**
Anthracene	**AN**	Dibenzo[a,h]pyrene	**DBahP**
Benz[a]anthracene	**BaA**	Dibenzo[a,i]pyrene	**DBaiP**
Benzo[a]pyrene	**BaP**	Dibenzo[a,l]pyrene	**DBalP**
Benzo[b]fluoranthene	**BbFA**	Fluoranthene	**FLT**
Benzo[c]fluorene	**BcFl**	Fluorene	**FLN**
Benzo[g,h,i]perylene	**BghiP**	Indeno[1,2,3-cd]pyrene	**IP**
Benzo[j]fluoranthene	**BjFA**	Phenanthrene	**PHE**
Benzo[k]fluoranthene	**BkFA**	Pyrene	**PY**
Chrysene	**CHR**		

Table S2: Specification of POP groups.

POPs group	
PCB	∑8 polychlorinated biphenyls congeners
DDT	∑2 dichlorodiphenyltrichloroethane isomers and their metabolites
HCB	hexachlorobenzene
HCH	∑3 hexachlorocyclohexane isomers
PBDE	∑7 polybrominated diphenyl ether congeners
HBCD	∑3 hexabromocyclododecane isomers
PBP	pentabromophenol
PFSA	∑4 perfluorinated sulfonates
PFCA	∑11 perfluorinated carboxylic acids
PAHs	∑23 polycyclic aromatic hydrocarbons
